# Overexpression of regenerating gene Iα appears to reflect aberration of crypt cell compartmentalization in sessile serrated adenoma/polyps of the colon

**DOI:** 10.1186/1746-1596-8-187

**Published:** 2013-11-13

**Authors:** Kentaro Okamoto, Takahiro Fujimori, Takeshi Yamaguchi, Kazuhito Ichikawa, Shigeki Tomita, Tamotsu Sugai, Johji Imura, Yasuo Ohkura, Takashi Yao, Shigehiko Fujii, Toshihiro Kusaka, Akira Sekikawa, Hirokazu Fukui, Tsutomu Chiba, Hiroyuki Kato, Hiroyuki Mitomi

**Affiliations:** 1 Department of Surgical and Molecular Pathology, Dokkyo Medical University School of Medicine, 880 Kitakobayashi, Mibu, Shimotsuga, Tochigi 321-0293, Japan; 2 First Department of Surgery, Dokkyo Medical University School of Medicine, 880 Kitakobayashi, Mibu, Shimotsuga, Tochigi 321-0293, Japan; 3 Division of Molecular Diagnostic Pathology, Department of Pathology, School of Medicine, Iwate Medical University, 19-1 Uchimaru, Morioka, Iwate 020-8505, Japan; 4 Department of Diagnostic Pathology, Graduate School of Medicine and Pharmaceutical Sciences, University of Toyama, 2630 Sugitani, Toyama City, Toyama 930-0194, Japan; 5 Department of Pathology, Kyorin University School of Medicine, 6-20-2 Shinkawa, Mitaka-shi, Tokyo 181-8611, Japan; 6 Department of Human Pathology, Juntendo University School of Medicine, 3-1-3 Hongo, Bunkyo-ku, Tokyo 113-8431, Japan; 7 Department of Gastroenterology, Kyoto Katsura Hospital, 17 Yamada Hirao, Nishikyo-ku, Kyoto 615-8256, Japan; 8 Department of Gastroenterology and Hepatology, Osaka Red Cross Hospital, 5-30 Fudegasaki, Tennoji-ku, Osaka 543-8555, Japan; 9 Division of Upper Gastroenterology, Department of Internal Medicine, Hyogo College of Medicine, 1-1 Mukogawa, Nishinomiya, Hyogo 663-8501, Japan; 10 Department of Gastroenterology and Hepatology, Kyoto, University Graduate School of Medicine, Yoshida-Konoe, Sakyo-ku, Kyoto 606-8501, Japan

**Keywords:** Colon, Crypt cell compartmentalization, Hyperplastic polyp, REG Iα, Sessile serrated adenoma/polyp

## Abstract

**Background:**

Colorectal sessile serrated adenoma/polyps (SSA/Ps) are characterized by asymmetrical distribution of Ki67-positive cells, which varies among crypts and involves the crypt length to a variable extent; the pattern has been designated as aberration of crypt cell compartmentalization. The *regenerating gene* (*REG*) Iα is a cell growth and/or anti-apoptotic factor and its overexpression might be associated with aberration of crypt cell compartmentalization in SSA/Ps. We investigated REG Iα expression in SSA/Ps in comparison to hyperplastic polyps (HPs).

**Methods:**

A total of 64 cases of serrated polyps (≥10 mm in size), including 53 SSA/Ps and 11 HPs, were included in the present study. Immunostaining was performed using a labeled streptavidin-biotin method. REG Iα expression was classified as follows: (i) expression of endocrine cells: grade 0 (a few positive cells) to 3 (marked increase in positive cells); (ii) expression of goblet cells: grade 0 (negative) to 2 (positive for crypts and surface epithelial cells); (iii) staining intensity of goblet cells: grade 0 (negative) to 2 (strong); (iv) staining intensity of crypt (absorptive) cell membranes: grade 0 (negative) to 2 (strong). The presence of aberration of crypt cell compartmentalization was assessed using Ki67 immunostaining.

**Results:**

With regard to the REG Iα expression of endocrine cells, 8 out of 11 HPs (73%) were grade 0, whereas 51 of 53 SSA/Ps (96%) were grade 1 or higher (*p* < 0.001). With regard to the distribution of REG Iα-immunoreactive goblet cells, 10 of 11 HPs (91%) were grade 1, whereas 50 of 53 SSA/Ps (94%) were grade 2 (*p* < 0.001). A similar trend was found in the staining intensity of goblet cells or crypt cell membranes (*p* = 0.011). Aberration of crypt cell compartmentalization was more frequently identified in SSA/Ps (72%) than in HPs (18%; *p* = 0.002). A significant association was observed between REG Iα overexpression and the aberration of crypt cell compartmentalization in serrated polyps (*p* = 0.037).

**Conclusions:**

REG Iα overexpression is a characteristic of SSA/Ps, which appears to reflect aberration of crypt cell compartmentalization.

**Virtual slides:**

The virtual slide(s) for this article can be found here: http://www.diagnosticpathology.diagnomx.eu/vs/7240956081100040

## Background

In 1996, Torlakovic and Snover were the first to describe sessile-type serrated adenoma of the colon in an analysis of serrated adenomatous polyposis with the development of adenocarcinoma [1]. In 2003, Torlakovic et al. [2] refined the criteria and proposed a subclassification of colorectal serrated polyps into hyperplastic polyps (HPs), traditional serrated adenomas, and sessile serrated adenomas. The last category of serrated polyps is now designated as sessile serrated adenomas/polyps (SSA/Ps) in the fourth edition of the WHO Classification of Tumors of the Digestive System [3]. Although SSA/Ps and large HPs (≥10 mm in diameter), the latter was introduced by Warner et al. in 1994 [4], were synonymously used at times [5], SSA/Ps were histologically separated from large HPs [6,7]. SSA/Ps were characterized by irregular and asymmetrical distribution of Ki67-positive cells, which frequently varied among crypts and involved the crypt length to a variable extent; the pattern was designated as “aberration of crypt cell compartmentalization” [8]. This is consistent with the results of computer-assisted cytometric analysis of Ki67 immunoreactivity in SSA/Ps [6]. However, aberration of crypt cell compartmentalization was not a characteristic of large HPs as well as conventional (small) HPs [6,7].

The regenerating gene (REG) Iα protein, the human homologue of the rat REG protein, was originally isolated from regenerating pancreatic islets [9], which were immunohistochemically positive for acinar, but not from islet cells of the pancreas [10]. REG Iα protein has been found to be expressed in normal colorectal mucosa and colorectal tumors [11]. REG Iα is also involved in the ulcerative colitis-neoplasia sequence [12,13]. Previous experimental studies have shown that REG Iα promotes cell growth and/or anti-apoptosis of cancer cells [12,14].

A link between REG Iα and β-catenin has been demonstrated in a study of liver cancer, in which β-catenin mutations induced REG Iα expression in liver cancer cells [15]. In this context, REG Iα expression together with aberrant β-catenin expression was associated with high Ki67 immunoreactivity in salivary gland tumors [16]. Recent studies have detected aberrant nuclear accumulation of β-catenin in SSA/Ps [17,18]. Wnt stimulation has been shown to lead to the inactivation of APC and the activation of β-catenin, resulting in nuclear accumulation of β-catenin, which subsequently complexes with the T-cell factor/lymphoid enhancer factor to activate target gene transcription resulting in cell proliferation [19]. These findings suggest that activation of Wnt/β-catenin signaling is associated with aberration of crypt cell compartmentalization in SSA/Ps.

The aim of this study was to investigate the expression of REG Iα in a subset of SSA/Ps and HPs and to discuss its expression in relation to nuclear β-catenin expression and aberration of crypt cell compartmentalization.

## Methods

### Tissue samples and histological examination

Samples of 154 cases of serrated polyps (≥10 mm in size) were obtained from the Dokkyo Medical University Hospital and its affiliated institutions between 2003 and 2010. The diagnosis of SSA/P was made when a serrated lesion had two or more of the following three findings: irregularly branching crypts (>10% of the lesion), horizontally arranged basal crypts (inverted T- and/or L-shaped crypts) (>10% of the lesion), and crypt dilatation (>10% of the lesion), according to a previous report [6]. Serrated polyps with only one of the three findings were designated as intermediate type, and this type was included in HPs. Criteria for HP included narrow straight crypts with a normal distribution of the proliferative zone at the base of the crypts with uniform maturation and serration toward the surface [3]. Cases of mixed serrated polyps and conventional adenoma or traditional serrated adenoma were excluded. Some of the serrated polyps were excluded because of poor orientation of the specimens. Finally, a total of 64 cases of serrated polyps, including 53 SSA/Ps and 11 HPs (histologically renamed large HPs), were included in the present study. Clinicopathological characteristics of the serrated polyps studied are summarized in Table 1.

**Table 1:** Clinicopathological characteristics of colorectal serrated polyps studied

	**SSA/P (n = 53)**	**HP (n = 11)**
Gender		
Male	28	6
Female	25	5
Age (yrs)^*^	55.4 (35–77)	59.3 (38–69)
Location		
Cecum	13	1
Ascending colon	16	3
Transverse colon	17	3
Descending colon	3	0
Sigmoid colon	4	3
Rectum	0	1
Tumor size (mm)^*^	13.2 (10–30)	10.6 (10–15)

^*^Age and tumor size are represented as mean (range).

SSA/P: sessile serrated adenoma/polyp; HP: hyperplastic polyp.

The ethics committee of the Dokkyo Medical University School of Medicine approved all protocols, and informed consent for tissue procurement was obtained from all patients. This work was conducted in a blinded manner using a linkable anonymizing method. Samples used in this study were materials obtained for diagnosis or treatment, but not for research purposes. Participation in the present study did not increase the medical disadvantage or risk for patients.

### Immunohistochemistry

Immunohistochemical staining for REG Iα and Ki67 was performed with an LSAB-2 kit (Dako, Glostrub, Denmark) as described previously [13,20]. In brief, 4-μm sections were placed on slides, deparaffinized, rehydrated, and then pretreated with 0.3% H_2_O_2_ in methanol for 20 min at room temperature to quench endogenous peroxidase activity. The slides were then placed in 0.01 ml/L citrate buffer (pH 6.0) and treated by microwave heating (400 W, 95°C; MI-77; Azumaya, Tokyo, Japan) to facilitate antigen retrieval.

The sections were first incubated with 1% bovine serum albumin in phosphate-buffered saline (PBS; pH 7.2) for 30 min and then with an anti-REG Iα antibody (the source of the antibody is described in reference [10]; dilution 1:2,000) and anti-Ki67 antibody (DAKO; dilution 1:50) for 1 h. Thereafter, the sections were incubated with a biotinylated secondary antibody for 15 min, washed with PBS, and treated with peroxidase-conjugated streptavidin for 20 min. Finally, the sections were incubated in 3, 3′-diaminobenzidine tetrahydrochloride (Liquid DAB + Substrate Chromogen System; Dako, USA) with 0.05% H_2_O_2_ for 3 min and then counterstained with Mayer’s hematoxylin.

### Evaluation of immunohistochemical staining

**Figure 1:**
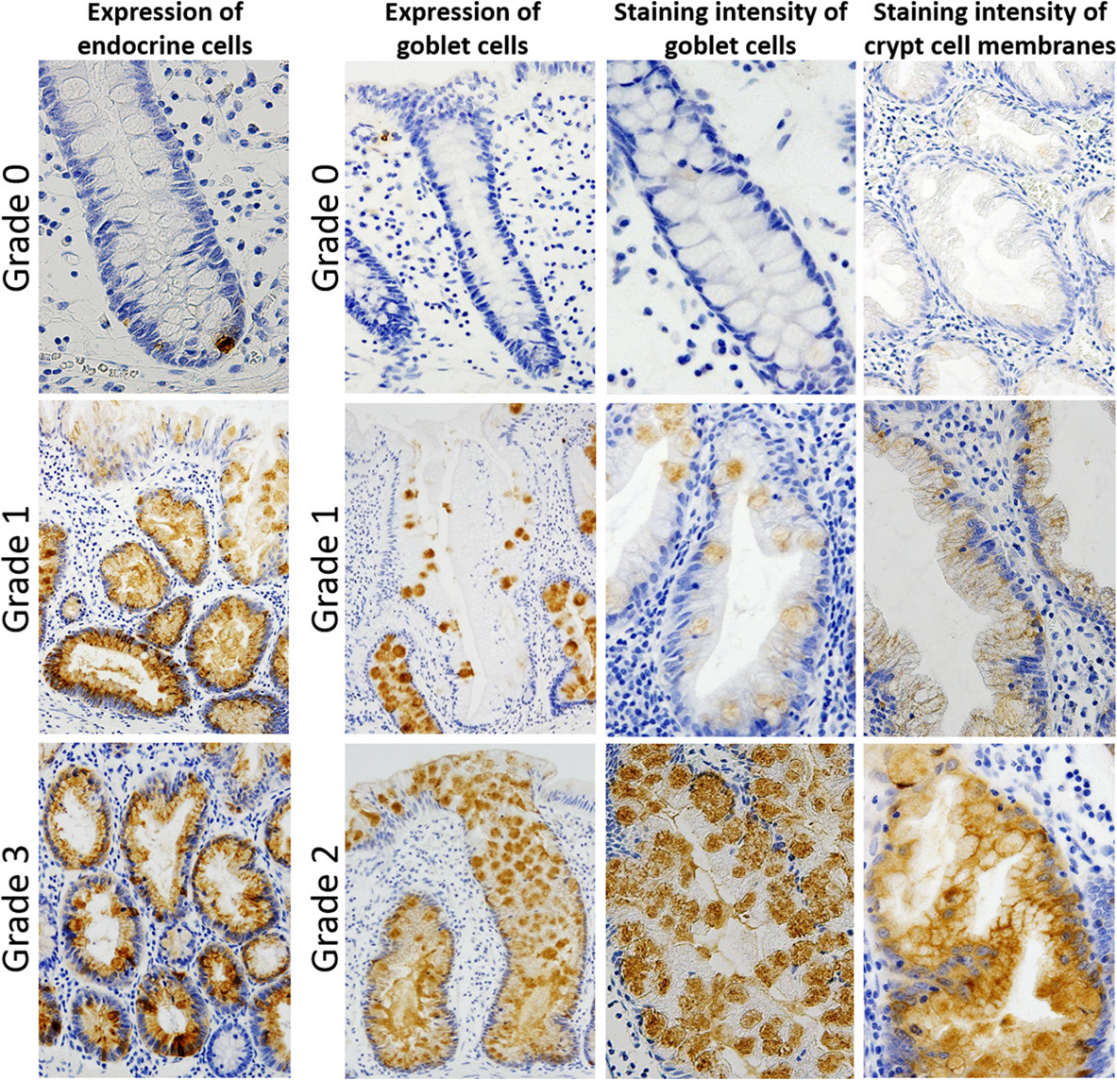
Immunohistochemical assessment (grading) of REG Iα expression.

In normal colon, REG Iα expression is found in goblet cells and endocrine cells [13]. Furthermore, by using immunohistochemistry for chromogranin A, REG Iα has been confirmed to be present exclusively in endocrine cells [21]. REG Iα positive endocrine cells are pyramidally shaped and closed with a broad base that directly abuts the basement membrane. We therefore classified the expression of REG Iα in a semiquantitative method according to the following scheme: (i) expression of endocrine cells: grade 0, a few (<1% of crypt cells) positive cells at the bottom of the crypt; grade 1, mild increase (1-5% of crypt cells) in the expression at the bottom of the crypt; grade 2, moderate increase (6-10% of crypt cells) in the expression at the bottom of the crypt and/or slight increase in the expression extending upward to surface epithelial cells; grade 3, marked increase (>10% of crypt cells) in the expression in crypt and surface epithelial cells; (ii) expression of goblet cells: grade 0, negative; grade 1, expression in crypt cells, but not in surface epithelial cells (≤50% of crypt cells); grade 2, expression in crypt and surface epithelial cells (>50% of crypt cells); (iii) staining intensity of goblet cells: grade 0, negative staining in goblet cells; grade 1, weak expression; grade 2, strong expression; (iv) staining intensity of crypt (absorptive) cell membranes: grade 0, negative expression in any epithelial cell membrane; grade 1, weak membranous expression; grade 2, strong membranous expression. Immunostaining was evaluated in most representative areas showing highest intensity. Representative microphotographs of REG Iα expression are illustrated in Figure 1.

We assessed the presence of aberration of crypt cell compartmentalization using Ki67 immunostaining and applied the criteria for aberration of crypt cell compartmentalization as described by Torlakovic et al. [8] as follows: an irregular distribution of Ki67-immunoreactive cells, which frequently varied among crypts, involved the crypt length to a variable extent, and was asymmetric in each individual crypt. Aberration of crypt cell compartmentalization was considered to be present when this feature existed in more than 10% of the crypt.

Every slide was examined simultaneously by four authors (KO, TY, HM, and TF) using a multi-head microscope, without prior knowledge of the clinicopathological data. In case of disagreement in the assessment of REG Iα expression and the presence of aberration of crypt cell compartmentalization, the slide was re-examined and consensus was reached on further review.

### Statistical analysis

Categorical analysis of variables was performed using either the chi-squared test (with Yates’ correction) or Fisher’s exact test, as appropriate. Continuous data were compared with the Mann-Whitney *U*-test. A *p* value of <0.05 was considered statistically significant. All statistical analyses were carried out using the R software (version 2.15.0).

## Results

**Figure 2:**
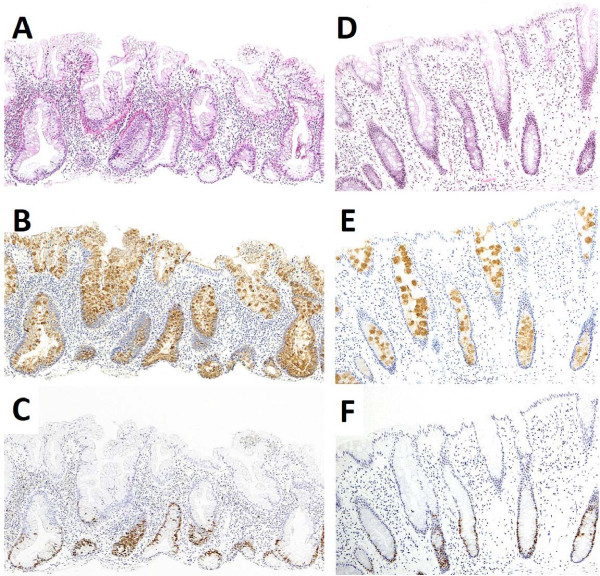
**Histology and immunohistochemistry for REG Iα and Ki67 of SSA/P and HP. (A)** SSA/P showing irregularly branching crypts, horizontally arranged basal crypts, and crypt dilatation (hematoxylin & eosin); **(B)** REG Iα is mainly expressed in goblet cells in the entire crypt and surface epithelium in SSA/P (immunoperoxidase); **(C)** Ki67-immunoreactive cells in SSA/P are either increased or decreased or both, with irregular distribution; **(D)** HP represents narrow-shaped crypts without luminal serration in the basal part (hematoxylin & eosin); **(E)** REG Iα is weakly and sparsely expressed in goblet cells in HP (immunoperoxidase); **(F)** Ki67-immunoreactive cells are distributed regularly in the basal part of crypts in HP.

**Figure 3:**
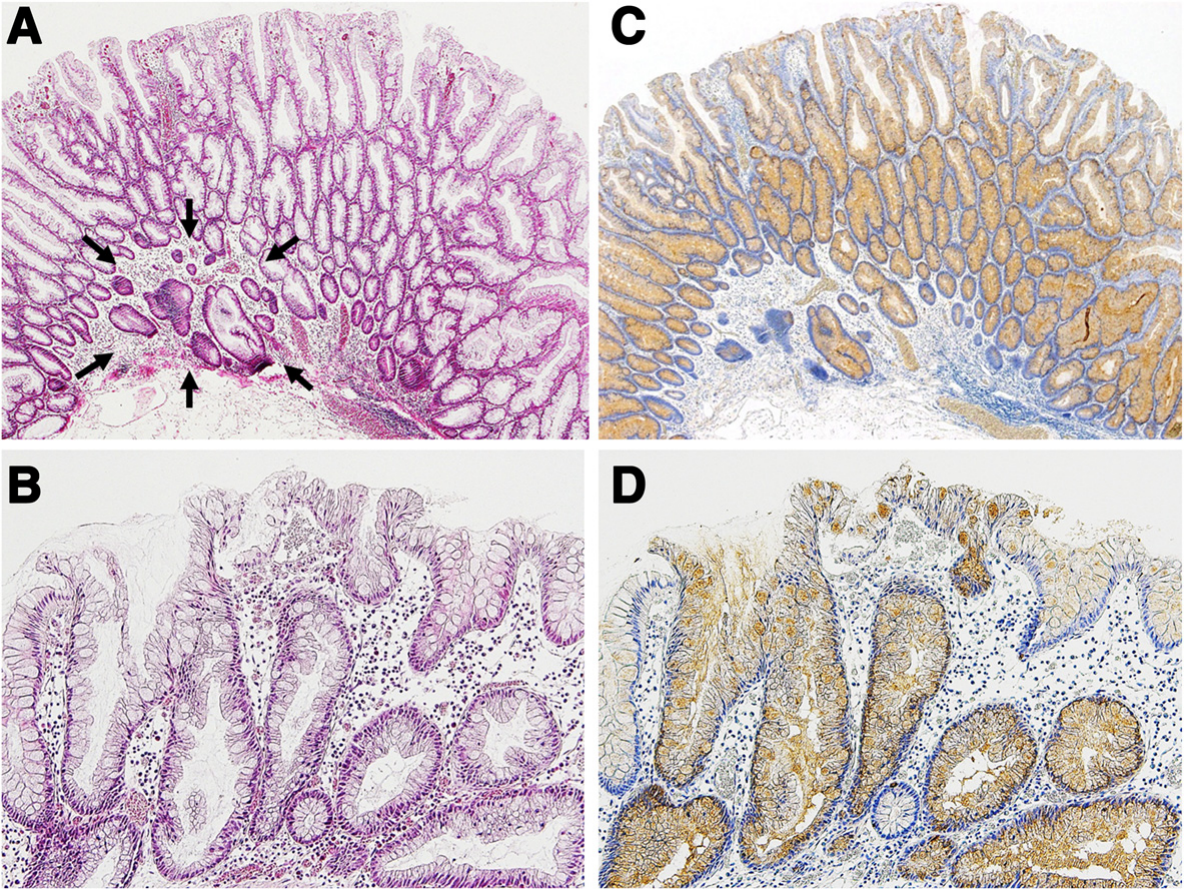
**A case of inverted HP in the ascending colon. (A)** Epithelial misplacement into the submucosa (*arrows*); **(B)** micropapillary projection at the surface area; **(C)** diffuse positivity for REG Iα in misplaced glands of the submucosa as well as in all glands of the mucosa; **(D)** REG Iα positivity representing goblet cells in the surface epithelium.

With regard to the REG Iα expression of endocrine cells, 8 out of 11 HPs (73%) were grade 0 and 51 of 53 SSA/Ps (96%) were grades 1 to 3 (*p* <0.001). With regard to REG Iα expression of goblet cells, 10 of 11 HPs (91%) were grade 1 and 50 of 53 SSA/Ps (94%) were grade 2 (*p* <0.001); a similar trend was found in the REG Iα staining intensity of goblet cells (*p* <0.001). None of the HP cases showed crypt cell membrane expression of REG Iα, but 26 of 53 SSA/Ps (49%) were positive for membranous expression (*p =*0.011; Table 2). The sum of the grading scores for the four items described above ranged from 2 - 9 (median, 6) in SSA/Ps and 2 - 4 (median, 3) in HPs; the values were significantly higher in SSA/Ps than in HPs (*p* <0.001). The expression patterns of REG Iα in representative cases of SSA/Ps and HPs are illustrated in Figure 2. In a case of HP, diagnosed as inverted HP by serial sections, REG Iα expression of goblet cells was grade 2 (Figure 3).

**Table 2:** Immunoreactivity of REG Iα in SSA/Ps and HPs

**REG Iα expression**	**SSA/P (n = 53)**	**HP (n = 11)**	***p *** **value**
Distribution of endocrine cells			<0.001
Grade 0	2	8	
Grade 1	30	1	
Grade 2	17	2	
Grade 3	4	0	
Distribution of goblet cells			<0.001
Grade 0	0	0	
Grade 1	3	10	
Grade 2	50	1	
Staining intensity of goblet cells			<0.001
Grade 0	0	0	
Grade 1	5	7	
Grade 2	48	4	
Staining intensity of crypt cell membrane			0.011
Grade 0	27	11	
Grade 1	19	0	
Grade 2	7	0	

SSA/P: sessile serrated adenoma/polyp; HP: hyperplastic polyp.

Aberration of crypt cell compartmentalization was more frequently identified in SSA/Ps (38 of 53 [72%]) than in HPs (2 of 11 [18%]; *p* =0.002). Of note, inverted HPs demonstrated aberration of crypt cell compartmentalization. In addition, we examined the association of REG Iα expression with aberration of crypt cell compartmentalization. In a combined analysis of SSA/Ps and HPs, high REG Iα expressors (sum of the grading score ≥5) were more frequent in serrated polyps with aberration of crypt cell compartmentalization (37 out of 40 [93%]) than in those without (14 out of 24 [58%]; *p* =0.037).

**Figure 4:**
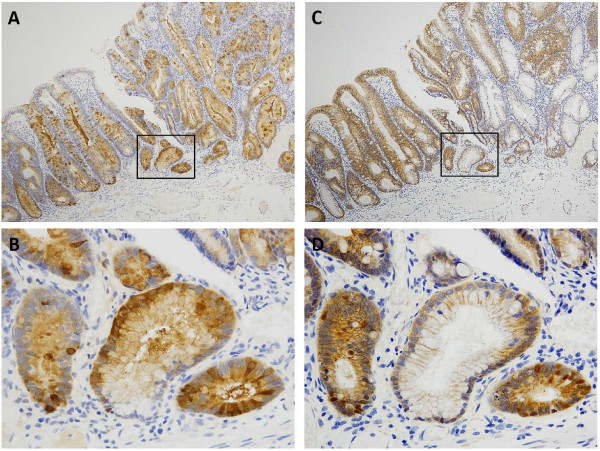
**Expressions of REG Iα and β-catenin in serial sections of a case of SSA/P. (A)** Diffuse REG Iα expression observed in the whole crypt cell population and surface epithelial cells; **(B)** strong cytoplasmic REG Iα staining mainly in cells at the bottom of the crypt (magnifying view of ‘box’ in figure **A**); **(C)** β-catenin is expressed mainly in the cell membrane, except at the bottom of the crypt; **(D)** note expression of nuclear and cytoplasmic β-catenin at the bottom of the crypt, similar to REG Iα (magnifying view of ‘box’ in figure **C**).

We performed an ancillary immunohistochemical study of β-catenin (monoclonal antibody, Transduction Laboratories, Lexington, KY; dilution 1:2,000) in a case of SSA/P (Figure 4). In this case, nuclear and cytoplasmic expression of β-catenin was evident at the bottom of the crypt, similar to REG Iα.

## Discussion

This is the first report to analyze REG Iα expression and crypt cell compartmentalization in serrated polyps. We show that REG Iα overexpression is a characteristic of SSA/Ps, as compared to (large) HPs. In analyses of ulcerative colitis-associated neoplasia, the expression of REG Iα gradually increased from regenerative mucosa through low-grade dysplasia to high-grade dysplasia; in this sequence, the distribution of proliferative cells increased similar to the REG Iα-positive region [12,13]. In addition, REG Iα-positive pleomorphic adenoma of the salivary gland demonstrated a significantly higher Ki67 labeling score than those negative for REG Iα [16]. In previous reports, SSA/Ps demonstrated high proliferative activity with asymmetric localization of proliferative cells [6-8]. This observation is in line with our results that aberration of crypt cell compartmentalization was more frequently identified in SSA/Ps (72%) than in HPs (18%). We also found a significant association between REG Iα overexpression and aberration of crypt cell compartmentalization in serrated polyps. In normal colonic crypts, endocrine cells and Paneth cells exist, in general, in proliferative and intermediate regions, and goblet cells are present only in the intermediate region [19]; however, in SSA/Ps, numerous goblet cells are identified at the base of the crypts (proliferative region) as well as in the intermediate region [2]. In SSA/Ps, there are abnormalities in the location of the various compartments (previously referred to as abnormal proliferation or dysmaturation [2]), a feature that Torlakovic et al. designated as the aberration of crypt compartmentalization [8].

Interestingly, we found that REG Iα-expressing endocrine cells are increased in SSA/Ps. To our knowledge, endocrine cell hyperplasia has not been previously described in the context of SSA/Ps. In microvesicular-type HP, the number and/or size of endocrine cells are increased and they are mainly found in the intermediate region of the crypts [2]. Recently, Naert et al. reported a case of large cell neuroendocrine carcinoma arising in an SSA [22]. Corresponding to this case and our findings, REG Iα is thought to be associated with endocrine cell hyperplasia and the development of neuroendocrine tumors in SSA/Ps. Mutations in *REG Iα* were identified in patients with carcinoid tumors [23], suggesting a link between its gain-of-function mutation and endocrine cell hyperplasia.

In our ancillary study, REG Iα positivity was related to aberrant (non-membranous type) β-catenin expression in SSA/Ps. In conjunction with this finding, aberrant β-catenin expression was related to REG Iα positivity in pleomorphic adenoma [16]. A link between REG Iα and β-catenin has been demonstrated in a study of liver cancer, in which β-catenin mutations induced REG Iα expression in liver cancer cells [15,16]. Therefore, REG Iα may be a possible downstream target of the Wnt/β-catenin signaling pathway. Wu et al. found aberrant nuclear labeling for β-catenin in 9 of 22 cases of SSA/Ps (41%). In a recent study, widespread or focal nuclear accumulation of β-catenin (using an N-terminus antibody) was also identified in 14 of 35 right-sided SSA/Ps (40%). Consequently, REG Iα overexpression may contribute to the early activation of the Wnt/β-catenin signaling pathway in SSA/Ps.

In the present study, we found one case of inverted HP displaying REG Iα overexpression in the ascending colon with a maximum diameter of 15 mm. This is the only case of HP with REG Iα overexpression similar to SSA/Ps. Inverted HPs as first described by Sobin et al. are an unusual morphological variant of HPs that show epithelial misplacement into the submucosa [24]. Inverted HPs are located in the rectum or sigmoid colon; their mean size is 5 mm [25]. To date, a small number of cases of inverted HP associated with adenoma and adenocarcinoma have been reported [26-28]. An association of SSA/Ps with inverted HPs in addition to their ability of malignant progression remains unknown and an area of research and controversy.

## Conclusions

This is the first report to demonstrate a correlation between SSA/P and REG Iα expression. REG Iα overexpression is a characteristic of SSA/Ps, which might be associated with aberration of crypt cell compartmentalization.

## Abbreviations

SSA/P: Sessile serrated adenoma/polyp; HP: Hyperplastic polyp.

## Competing interests

No financial and non-financial competing interests to declare in relation to this manuscript.

## Authors’ contributions

Study concept and design: TF and KO; immunohistochemical analysis: TY, KI ST and JI; acquisition of data: SF, TK, AS and HF; analysis of interpretation of data: HM and KO; drafting of the manuscript: KO, TH and HM; critical revision of the manuscript for intellectual content: TS, YO, TY and TC; study supervision: HK and TF. All authors read and approved the final manuscript.
